# Particular difficulties faced by GPs with young adults who will attempt suicide: a cross-sectional study

**DOI:** 10.1186/1471-2296-14-68

**Published:** 2013-05-24

**Authors:** Nadia Younes, Christine Chan Chee, Clement Turbelin, Thomas Hanslik, Christine Passerieux, Maria Melchior

**Affiliations:** 1 EA 40–47 Université Versailles Saint-Quentin-en-Yvelines, Versailles F-7800, France; 2 Academic Unit of Psychiatry, Versailles Hospital, Le Chesnay F-78157, France; 3 French Institute for Public Health Surveillance, Saint Maurice, France; 4 INSERM, U707, Paris F-75012, France; 5 UPMC Université Paris 06, UMR-S U707, Paris F-75012, France; 6 Université Versailles Saint Quentin en Yvelines, Versailles F-78000, France; 7 UMRS 1018, Université de Versailles Saint-Quentin en Yvelines, Hôpital Paul-Brousse, 16 avenue Paul Vaillant-Couturier, Villejuif F-94807, France; 8 INSERM U1018, Centre for research in Epidemiology and Population Health, Epidemiology of occupational and social determinants of health, Hôpital Paul-Brousse, 16 avenue Paul Vaillant-Couturier, Villejuif F-94807, France

**Keywords:** Young adults, General practice, Suicidal attempt, Prescription, Referral

## Abstract

**Background:**

Suicide is a major public health problem in young people. General Practitioners (GPs) play a central role in suicide prevention. However data about how physicians deal with suicidal youths are lacking. This study aims to compare young adult suicide attempters (from 18 to 39 years old) with older adults in a primary care setting.

**Methods:**

A cross-sectional study was carried. All suicide attempts (N=270) reported to the French Sentinel surveillance System from 2009 to 2011 were considered. We conducted comparison of data on the last GP’s consultation and GPs’ management in the last three months between young adults and older adults.

**Results:**

In comparison with older adults, young adults consulted their GP less frequently in the month preceding the suicidal attempt (40.9 vs. 64.6%, p=.01). During the last consultation prior to the suicidal attempt, they expressed suicidal ideas less frequently (11.3 vs. 21.9%, p=.03). In the year preceding the suicidal attempt, GPs identified depression significantly less often (42.0 vs. 63.4%, p=.001). In the preceding three months, GPs realized significantly less interventions: less psychological support (37.5 vs. 53.0%, p=.02), prescribed less antidepressants (28.6 vs. 54.8%, p<.0001) or psychotropic drugs (39.1 vs. 52.9%, p=.03) and made fewer attempts to refer to a mental health specialist (33.3 vs. 45.5%, p=.05).

**Conclusion:**

With young adults who subsequently attempt suicide, GPs face particular difficulties compared to older adults, as a significant proportion of young adults were not seen in the previous six months, as GPs identified less depressions in the preceding year and were less active in managing in the preceding three months. Medical training and continuing medical education should include better instruction on challenges relative to addressing suicide risk in this particular population.

## Background

Worldwide, suicide is the second leading cause of death in young people, representing a major public health problem [1]. Several studies report especially high rates of suicide attempts in young people: in the 20–24 year age group among men (427 (95% CI: 416–439) per 100,000) and in the 15–19 year age group among women (620 (95% CI: 605–636) per 100,000) [2,3]. Suicidal attempt is the strongest risk factor for suicide, psychiatric disorders which tend to onset in young adulthood being the second [4]. Other contributors include genetic vulnerability, psychological, familial, social, and cultural factors [5].

There is no consensus regarding the definition of young adults. For example, Gulliver et al. proposed to differentiate ‘adolescents’ (aged between 12 and 17 years) from ‘young adults’ (between 18 and 25 years) [6]. Other authors considered that young people include anyone aged 15 to 25 [7,8], 16 to 20 [9], 15 to 34 [10], 18 to 34 [11,12] or 15–39 [13]. In the present investigation, we considered young adults ages 18 to 39, following Erik Erikson’s widely cited life model and Daniel Levinson’s model, to consider young adulthood in a developmental perspective [14,15]. It allowed us to exclude adolescents and to have a population with a significant rate of incident mental health difficulties.

Mental health access for young people is especially unsatisfactory: only 18 to 34% of young people with high levels of symptoms of depression or anxiety seek professional help [6]. In the ESEMED survey conducted in Europe, compared to participants older than 65, youths aged 18 to 24 were least likely to use formal health services or specialized care in case of mental health problems, and youths aged 25 to 34 third least likely [16]. For mental health problems, young people first turn to someone they know: friends and family followed by the General Practitioner (GP) [17]. When they access the health care for mental health reasons, it is first through primary care [7,18]. Young people with unmet psychological needs are not generally excluded from the healthcare system: 75 to 90% consult their GP at least once a year, in most cases for somatic complaints [9]. Thus, GPs have a unique opportunity to investigate mental health issues. About half of patients who attempt suicide (including young people) have visited a GP in the month preceding the attempt [13,19]. However stigmatization in young people towards people with mental disorders is still high [20]; others barriers exist in young people and in health care system, starting with GPs. Only one study explored the difference between young and older adults’ contacts with GPs before suicide attempt and revealed that older adults have higher rates of contact with primary care providers than younger adults within one month prior to suicide [12]. Recognition of potentially suicidal patients and their management by the GP are thus very challenging. Moreover data about how physicians deal with young adult suicidal patients are lacking.

The aim of this study was to compare GPs’ management of young adults (ages 18 to 39) and older adults (age 40 and above) who have subsequently attempted suicide in a primary care setting, as close as possible to the “real-world” experience.

## Methods

A cross-sectional study was carried.

### Data

We studied patients who attempted suicide as reported by their GP to the French Sentinel General Practice network (FSN). Study authorization was given from the scientific board of the FSN. The network consists of a sample of approximately 500 GPs all over metropolitan France, who continually report on an unpaid volunteer basis the occurrence of well-defined health related events in their daily practice [21]. Data are collected via an online survey. The surveillance of suicide (fatal) and of suicide attempt (non-fatal) was introduced in 1999. A suicide attempt is defined according to the WHO/EURO para-suicide definition as “an act of self-inflicted injury (for example, self-mutilation, jumping from high places, hanging, gunshots, asphyxiation) or self-poisoning with drugs in excess of the generally recognized therapeutic dose [22,23]. GPs are instructed to report all cases they were confronted with in their daily practice (age 18 and above without a superior limit), including persons seen while on duty and their own patients seen by other caregivers (emergency rooms mainly). For each suicidal event that occurred from 2009 to 2011 (suicide and suicidal attempt), GPs are instructed to report sociodemographic and clinical descriptions were completed with data about the last GP consultation (time, expression of suicidal ideation) and about the GP’s management of the patient: recognition in their practice (without a standardized tool) of “psychological difficulties” or of the specific diagnosis of “depression” in the last year (as depression is mainly associated with suicide risk), interventions with provision of psychological support, with psychotropic drugs prescription, with attempt to refer to a psychiatrist and with specialized care with a psychiatrist or with a psychologist in the three months preceding the suicide attempt.

### Analysis

We compared young adult suicidal attempters (ages 18 to 39 years) with older adult suicidal attempters (age 40 years and above), using Chi-square tests, Fisher’s exact test for categorical characteristics and Student *t* test for continuous covariates. Analyses were conducted using the Stata statistical software, version 11.0.

### Ethics statement

French Sentinelles network was approved by the National Data Protection Agency (CNIL, registration number #47139).

## Results and discussion

### Results

From January 2009 to December 2011, 270 suicide attempts were reported to the Sentinel network. Comparisons between young adults and older adults are presented in Table 1.

**Table 1:** Comparison of young adults (18–39 yrs old) and older adults (40 yrs and above) who attempted suicide (Sentinelle network, France, N= 270)

	**18-39 years old**	**>= 40 years old**	***Chi2 test p***
	**N=106**	**N=164**	
	**N (%)**	**N (%)**	
Demographics N (%)			
female	60 (56.6)	99 (60.4)	ns
male	45 (42.5)	63 (38.4)	
Time of the last consultation N (%)			0.02
< 1 week	25 (23.6)	41 (25.0)	
1-4 weeks	27 (25.5)	65 (39.6)	
1-6 months	32 (30.2)	43 (26.2)	
> 6 months	19 (17.9)	11 (6.7)	
Unknown	3 (2.8)	4 (2.4)	
Suicidal ideas expressed at the last consultation N (%)	12 (11.3)	35 (21.9)	0.03
Recognition by the GP of psychological difficulties in the last year N (%)			
Depression	42 (42.0)	97 (63.4)	0.001
Psychological difficulties	71 (68.3)	112 (74.7)	ns
GP’s management in the last three months (%)			
Psychological support	39 (37.5)	80 (53.0)	0.02
Antidepressant prescription	30 (28.6)	85 (54.8)	<0.001
Other psychotropic drugs prescription	41 (39.1)	83 (52.9)	0.03
Attempt to referral to a mental health specialist	34 (33.3)	70 (45.5)	0.05
Parallel care with a psychiatrist	33 (32.7)	64 (40.5)	ns
Parallel care with a psychologist	22 (21.6)	28 (17.4)	ns

Young and older suicide attempters did not statistically differ in terms of gender.

Young suicide attempters last consulted a GP less recently than older suicide attempters: 40.9% in the last month (versus 64.6% of older adults), 17.9% earlier than 6 months (vs 6.7% of older adults), 30.2% consulted between 1 to 6 months (vs 26.2%), p=.01.

During the last consultation prior to the suicidal attempt, Younger suicide attempters expressed suicidal ideas less frequently than their older counterparts (11.3% vs. 21.9%, p=.03) even after taking into account the time of the last consultation: when the last consultation took place in the last month 17.3% of young adults vs. 21.4% of older adults expressed suicidal ideas (p=.55) and respectively 5.9% vs. 20.4% (p=.03) when the last consultation was more than a month prior.

In the year preceding the suicidal attempt, GPs identified depression significantly less often in young than in older adults (42.0% vs. 63.4%, p=.001), but they identified psychological difficulties in about seven out of ten suicidal patients, without significant difference between young and older adults.

In the three months preceding the suicide attempt, GPs realized significantly fewer interventions among young adults: less psychological support (37.5% vs. 53.0%, p=.02), fewer antidepressant prescriptions (28.6% vs. 54.8%, p<.0001), fewer other psychotropic drug prescriptions (39.1% vs. 52.9%, p=.03) and referred their patients less to a mental health specialist (33.3% vs. 45.5%, p=.05). More than a third of the patients had had specialized care with a psychiatrist in addition to primary care and about two out of ten obtained specialized care with a psychologist, without significant difference between young and older adults.

### Strengths and limitations of the study

Sentinel General Practice network is a precious source of national clinical data about the last GP’s consultation and GPs’ management prior to suicide attempt in general practice.

Limitations should be acknowledged. We were not able to know if any suicide cases were missed by the Sentinel network. Overall, it is likely that non fatal suicidal events are underreported [24-27]. Participation data are however comparable to data obtained by the two other existing Sentinel Networks of General Practice (Belgian and Dutch) [25,28]. It should also be acknowledged that Sentinel GPs may not reflect practices for French GPs, since their involvement in the Network and in public health may make them more aware of certain problems such as suicide attempts.

Information provided by GPs may have been biased as the GP had knowledge of the suicide attempt at the time of documentation [29]. Information on participants’ mental health was also limited, since GPs identified psychological difficulties and depression without a validated instrument. In the future, it will be important to validate GPs’ perceptions of their patients’ mental health against validated measure instruments and to follow-up the risk of suicide in relation to psychological difficulties in prospective study designs. We were not able to consider others factors which could be related with differences in self-harm rates – for example, suicide attempts in younger adult may be more spontaneous, or linked to acute drug or alcohol misuse or to life events, whilst those in older adults may reflect chronic physical or mental illness and be associated with more planning and therefore time for help-seeking behaviour. We were also not able to consider the reason for attending the GP which may have affected the results: consultations for mental health are more likely to lead to disclosure of psychological distress than those for a somatic reason. Finally, the interpretation of results should take into account that information was collected at different time frames as GP were instructed to report data about the last consultation (time and suicidal ideas expression) (even if it was more than 6 months before), about their recognition of depression or psychological difficulties in the last year and about their management in the three months preceding the suicide attempt.

### Comparison with existing literature

Contacts before suicide attempt. Our results indicate that young adults consult GPs significantly less than older adults in the month preceding a suicide attempt. “This finding is contradictory with that of a large study in Finland conducted among suicide attempters treated in general hospitals, which found no major differences between the three age-groups (15–24, 25–39 or 40 and above) before the attempt in treatment contacts (without data on contacts with private health care providers) [13]. The difference in the settings (our data considered primary care suicide attempters) can explain the discrepancies between the two studies. It is not surprising that older people with their significant larger burden of physical health burdens are consulting GPs more. It is coherent with the review of evidence on contacts with primary care providers before suicide [12]. There is still considerable scope for improvement in access to care for young people who have psychological difficulties and are at risk of suicide and also reluctant to seek professional mental health care [17]. Prevention actions are difficult, especially among working-age groups [30,31]. Efforts have targeted seekers of specialty treatment at high risk of suicide, but fewer interventions have focused on individuals with suicidal thoughts or attempters who do not seek treatment, as men, 18- to 25-year-olds [32]. Public awareness campaign should be completed with physicians’ actions [33].

Suicidal ideas expression. When they accessed GP care (and it is a minority as above discussed), only 9.2% of young adults who later attempted suicide expressed suicidal ideas. These percentages are similar to those reported in previous studies of adults in primary care settings [19,25]. Studies conducted directly among suicidal patients indicate that only a minority felt suicidal at the last consultation and even fewer reported this to their GP [19,34]. GPs should also be aware of their own barriers in the exploration of suicidal ideas [35-37], and that the presenting problem could be convoluted [19,35]. The difference between young and old adults does not lie in fewer suicidal ideations in the former group: to the contrary, young adults have a high prevalence of suicide ideation [38] but may have difficulty expressing this. Young people’s intentions to seek help decrease as their level of suicidal ideation increases and the most commonly experienced mental health problems increase their preference to keep their distress to themselves [17,39]. GPs need to adopt a friendly attitude and systematically include questions on mental health including suicidal ideas and tackle taboo issues about emotions and feelings, in order to increase the number of young adults who disclose their psychological problems [6,9].

GP’s management prior to suicide attempt. GPs identified psychological difficulties in general as frequently in young rather than in older adult suicide attempters, but they identified depression less frequently in youngs. This is not explained by differences in depression prevalence. The prevalence of psychiatric disorders is higher among young people aged 16–24 years than in any other age group [6]. According to the National Comorbidity Survey Replication in the US, the median age of onset is 11 years for anxiety disorders and for impulse-control disorders (e.g. conduct disorder, attention deficit disorder), 20 years for substance use disorders, and 30 years for mood disorders [4,40]. Hickie summarized epidemiological evidence of this topic in the following way: 75% of the adult psychiatric disorders start before age 25. Up to 25% of young people will experience a mental health or substance-related problem in adolescence or early adulthood, and psychiatric disorders account for 60% of health-related disability in individuals aged between 15 and 34 years [40]. Some authors argue that young people’s health and wellbeing may have declined over several generations [41]. Less identification of depression among young adults is more plausible, reaching GPs’ difficulties to identify such state among young adults [42,43]. The recognition of existing psychological problems often depends on parental expressions of concern [44], as young adults are often reluctant to mention their personal emotional problems, because of more concerns about being viewed as weak or abnormal [17]. Stigmatization in young people towards people with mental disorders is still high [20]. It implies a GPs’ friendly attitude toward mental health issues and some active exploration. An experimental technique has been proposed in general practice for identification of depression in young people and appeared usable in routine practice, if used selectively [45]. It is challenging because early onset depression (before 40 years) is more severe than late onset depression [46].

The less active management of young adult patients by the GPs could in part be the consequence of the weaker identification of psychiatric disorders. GPs do less psychological support, prescribe fewer antidepressants or psychotropic drugs and make fewer attempts to refer young adult patients to a mental health specialist. This may result from a lesser sensitivity to psychological difficulties in young adults, implying that specific medical training and continuing medical education on this topic could enhance GPs screening and management skills. Fewer prescribing patterns of psychotropic drugs by primary care physicians has been documented in younger depressed adults [47]. Antidepressant treatment is also less likely to be continued in patients under 35 years, relatively to olders [48].

Collaborative care with mental health specialists (psychiatrists or psychologists) was comparable in the management of young and older adults in this study while in other studies, younger age has been associated with more referral [49-51]. Shared mental health care between primary and specialized care is a way to manage the reluctance of young people to seek professional help for mental health problems [52,53].

## Conclusions

With young adults who subsequently attempt suicide, GPs face particular difficulties compared to older adults: a significant proportion of young adults were not seen in primary care in the previous 6 months, GPs identified less depressions in the preceding year and were less active in managing in the preceding three months. Suicidal attempts could also be more opportunistic than in older adults. On the other hand, medical training, continuing medical education and collaborative care should include better instruction about challenges in identifying and addressing the mental health needs of young adults, which has been considered as “a new frontier in the health and social policy of the 21st century” [54]. Future research on this specific population in primary care should be conducted.

## Abbreviations

GP: General practitioner.

## Competing interests

The authors declare that they have no competing interest.

## Authors’ contributions

NY conceived of the study, participated in its design and drafted the manuscript. CCC participated in the design of the study, performed the statistical analyses. TC participated in the design and coordination of the study; he belongs to the statistical team in charge of the FSN. HT participated in the design of the study, as the medical director of the FSN. CP participated in the conception of the study. MM participated in the design and in the analyses of the study. All authors read and approved the final manuscript.

## Pre-publication history

The pre-publication history for this paper can be accessed here:

http://www.biomedcentral.com/1471-2296/14/68/prepub
